# Genetic analysis of the *GRIK2 *modifier effect in Huntington's disease

**DOI:** 10.1186/1471-2202-7-62

**Published:** 2006-09-07

**Authors:** Wenqi Zeng, Tammy Gillis, Michael Hakky, Luc Djoussé, Richard H Myers, Marcy E MacDonald, James F Gusella

**Affiliations:** 1 Molecular Neurogenetics Unit, Center for Human Genetic Research, Massachusetts General Hospital, 185 Cambridge Street, Boston, MA 02114, USA; 2 Evans Department of Medicine, Section of Preventive Medicine and Epidemiology, Boston University School of Medicine, Boston, MA 02118, USA; 3 Department of Neurology, Boston University School of Medicine, 715 Albany Street, Boston, Massachusetts, MA 02118, USA; 4 HDSA Coalition for the Cure Mitochondria and Energy Metabolism Team, USA

## Abstract

**Background:**

In Huntington's disease (HD), age at neurological onset is inversely correlated with the length of the CAG trinucleotide repeat mutation, but can be modified by genetic factors beyond the *HD *gene. Association of a relatively infrequent 16 TAA allele of a trinucleotide repeat polymorphism in the *GRIK2 *3'UTR with earlier than expected age at neurological onset has been suggested to reflect linkage disequilibrium with a functional polymorphism in *GRIK2 *or an adjacent gene.

**Results:**

We have tested this hypothesis by sequencing all *GRIK2 *exons, the exon-flanking sequences and 3'UTR in several individuals who were crucial to demonstrating the modifier effect, as they showed much earlier age at neurological onset than would be expected from the length of their HD CAG mutation. Though ten known SNPs were detected, no sequence variants were found in coding or adjacent sequence that could explain the modifier effect by linkage disequilibrium with the 16 TAA allele. Haplotype analysis using microsatellites, known SNPs and new variants discovered in the 3'UTR argues against a common ancestral origin for the 16 TAA repeat alleles in these individuals.

**Conclusion:**

These data suggest that the modifier effect is actually due to the TAA repeat itself, possibly via a functional consequence on the *GRIK2 *mRNA.

## Background

Huntington disease (HD [MIM 143100]) is a neurodegenerative disorder caused by an expanded CAG trinucleotide repeat that lengthens a polyglutamine tract near the amino terminus of the huntingtin protein [1]. The mutation involves a "gain-of-function" that leads to the selective loss of vulnerable neurons, most notably medium spiny neurons in the caudate nucleus, and results in characteristic progressive writhing movements, in addition to psychological changes and cognitive decline [2]. The age at onset of HD is variable, though the motor disturbance typically begins in mid-life, and is followed by an inexorable decline that ends in death after a course of 10–20 years. The length of the expanded CAG trinucleotide tract is the primary determinant of disease onset, although the mechanism whereby mutant huntingtin triggers the cascade of HD pathogenesis that eventually produces these symptoms is not yet clear [3,4]. Like several other polyglutamine neurodegenerative disorders, there is a strong inverse correlation between the length of the polyglutamine tract and the age at neurologic onset, with the longest mutations leading to juvenile HD. Indeed, the HD CAG repeat length alone accounts for more than half of the overall variance in age at neurologic onset. We and others have showed that the remaining variation in age at neurologic onset of HD is highly heritable and that other genetic factors act to modify the pathogenic process [5-16]. Identification and characterization of such modifiers is of critical importance, since their capacity to alter pathogenesis would make them potential targets for development of therapeutics for this currently untreatable disorder.

To date, one candidate genetic modifier of HD has been confirmed in multiple independent studies. An association between genetic variation at *GRIK2 *(GenBank: mRNA NM_021956), encoding the GluR6 subunit of the kainate receptor, and age at neurologic onset in HD was first reported by Rubinsztein et al., based on 293 HD patients from the United Kingdom [13]. A TAA repeat polymorphism (*D6S1028*; CHLC.ATA22H10) in the 3' untranslated region (UTR) of *GRIK2 *accounted for 13% of the residual variance in onset age after accounting for the effect of the HD CAG mutation. The 16 repeat allele (designated '155' based on the PCR product length) was found in those with younger than expected HD onset ages, leading to the suggestion that the TAA repeat was a neutral polymorphism in linkage disequilibrium with a functional variant in *GRIK2*, or in a nearby gene. The *GRIK2 *association was confirmed in 258 unrelated U.S. HD patients, where the relatively rare 16 TAA allele was associated with an approximate 5-year-younger age at onset [11]. Subsequently, a modifier role for this locus has been demonstrated in HD populations from France, India, and Italy [6,7,12]. To address the issue of a functional variant at or near the *GRIK2 *locus, we have tested whether a coding sequence variant explains the modifier effect, and subsequently, we have used analysis of polymorphisms in the *GRIK2 *region to implicate the TAA repeat itself as the source of the genetic modification of onset age.

## Results

### Testing for a functional coding sequence variant in *GRIK2*

The *GRIK2 *TAA repeat displays at least 8 alleles, ranging from 10 to 17 TAA units, though more than 90% of chromosomes have 13, 14 or 15 repeats. The largest alleles, 16 and 17 TAAs, make up only 3.1% and 0.3% of chromosomes in our sample (n = 622), respectively. Our original confirmation of the *GRIK2 *modifier effect employed 258 unrelated affected individuals of known onset available to the Massachusetts HD Center Without Walls and did not account for subsequently defined effects of the normal HD CAG allele and interaction between the mutant and wild-type CAGs [11]. We have now expanded this test population to 311 individuals, genotyped for both the *HD *CAG repeat and the *GRIK2 *TAA repeat. The variability in onset age attributable to the *HD *CAG repeat, wild-type HD CAG and interaction of the two [ln (age at onset) = HD CAG repeat + normal CAG repeat + (HD CAG repeat)*(normal CAG repeat)] is *R*^2 ^= 0.743. When the presence of a 16 TAA repeat allele is added to the model, a statistically significant amount of the variability in onset age is explained: *R*^2 ^= 0.748, (16 allele genotypes, *p *= 0.02). Those genotypes that include a 16 allele have a mean age at onset approximately 4.5 years younger than persons with only other *GRIK2 *alleles.

On the basis of these data, we selected three individuals who possessed a 16 TAA allele and displayed much earlier onset of HD than expected based upon their respective CAG repeats. GUS17507, GUS3225 and GUS17508, had CAG repeats of 45, 46 and 47 units, respectively, which would typically be associated with onset between 50 and 58 years of age (Table 1). However, they displayed onset of neurologic symptoms at 33, 28 and 29 years, respectively. These three individuals contributed strongly to the detection of the modifier effect above, since leaving them out of the analysis dramatically altered the *p *value for the effect (*p *= 0.13). We also selected one individual, GUS17843 with a rare 17 TAA allele, a HD CAG repeat of 46 and an age at onset of 33. For comparison, we also chose four individuals with equivalent CAG repeats but a more typical age at neurologic onset. None of these four, GUS21069, GUS2433, GUS20708 and GUS18027, who had CAG repeats of 45, 46, 46, and 47 units and exhibited neurologic onset at 58, 57, 50 and 54 years, respectively, possessed either a 16 or 17 *GRIK2 *TAA allele (Table 1). To identify a functional polymorphism associated with the early onset phenotype, we sequenced the exons and exon boundaries for *GRIK2 *in all of these individuals.

**Figure 1:**
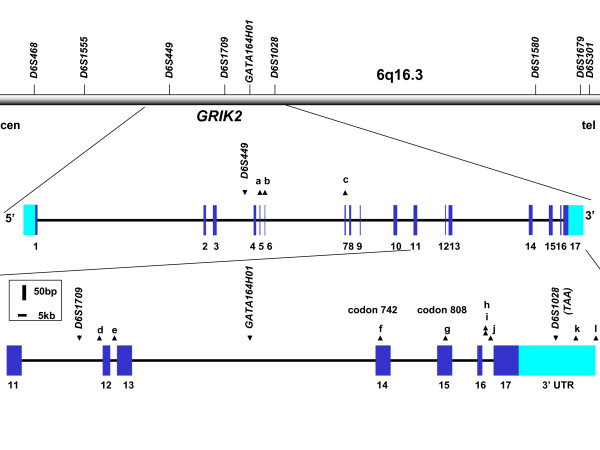
**Genomic structure of *GRIK2 *and locations of microsatellites and SNPs**. The relative positions of nine microsatellites chosen using the Human Genome Database and the UCSC Genome Browser are shown above a bar, oriented from centromere (cen) to telomere (tel), representing the chromosome 6q16.3 region containing *GRIK2 *[18, 33]. The *GRIK2 *gene, comprising 17 exons (numbered) is expanded below the bar, and the region from exon 11 through the 3'UTR is further expanded below. The annotation of the 3'UTR region of *GRIK2 *in human genome databases is incomplete, indicating only a shorter transcript associated with use of a polyadenylation signal upstream from the TAA repeat. Data from [29] indicate that two polyadenyation signals may be used, producing either a shorter mRNA, without the TAA repeat, or a longer mRNA that contains it. Coding sequences are shown in dark blue and UTRs are shown in light blue, with introns shown as a thin black bar. The locations of relevant microsatellites and SNPs are denoted by downward and upward-pointing triangles, respectively. SNPs located in the *GRIK2 *gene are denoted by letter: a: rs2852565, b: rs2786251, c: rs6922753, d: rs2518283, e: rs2243355, f: rs3213607, g: rs2227283, h: rs2852620, i: rs1034254, j: rs12198351, k: rs28383483, l: rs28383484. The last polymorphism is located at the edge of the region of extended homology with rodent mRNAs (see Figure 2) and may lie either at the extreme end of the human 3'UTR or immediately outside of it.

*GRIK2 *comprises 17 exons spanning ~670 kb in 6q16.3, with alternative splicing capable of producing alternative carboxyl termini for the protein (Figure 1). Sequencing of all exons revealed no coding sequence or boundary changes specific to patients with earlier than expected HD onset. In the eight HD individuals, we identified polymorphisms in the flanking sequences of exons 6 (2 SNPs), 7, 12, and 13, and in the coding sequences of exons 14 and 15, corresponding to known SNPs rs2852565, rs2786251, rs6922753, rs2518283, rs2243355, rs3213607, and rs2227283, respectively (Table 2). Neither of the coding sequence variants is predicted to alter an amino acid. The only other *GRIK2 *coding sequence SNPs reported in dbSNP are rs3213608 and rs2235076, which are predicted to cause V>I and M>I changes in exons 14 and 17, respectively [17] We did not detect either of these in any of the 8 individuals tested.

### Testing for a common origin of the alleles associated with younger onset

In the absence of a coding sequence variant, we next tested whether the 16 TAA allele might be in linkage disequilibrium with a functional variant in noncoding sequence or in an adjacent gene. This would predict that the particular chromosomes associated with the early onset phenotype would share a common ancestral origin. Consequently, we typed 8 microsatellite markers across the *GRIK2 *locus. These locations are shown in Figure 1, with the genotypes for the individuals tested in Table 2. Markers upstream from *GRIK2 *and in introns 3, 11 and 13 gave no evidence for a common haplotype shared among the individuals with a 16 TAA allele. Indeed, for the markers in introns 11 and 13, nearest the 3'UTR, at best two of the 3 individuals (GUS3223 and GUS17508) could have shared a two marker haplotype, '160' at *D6S1709 *(AFMA070ZD9) and '216' at GATA164H01. However, genotyping of the mother and a sibling of GUS3225 revealed that the 16 TAA allele was in coupling with the alternate GATA164H01 allele, 220 (data not shown). These data suggest that if the chromosomes bearing a 16 TAA allele and capable of modifying onset age share a common origin, the shared ancestral region would be 3' to the markers in intron 13. Combining these data with the SNP genotypes described above suggests further that a segment shared by the 16 allele chromosomes would be 3' to the missense SNP in exon 14, as GUS3225 and GUS17507 differ at this site on the 16 TAA allele chromosome.

**Figure 2:**
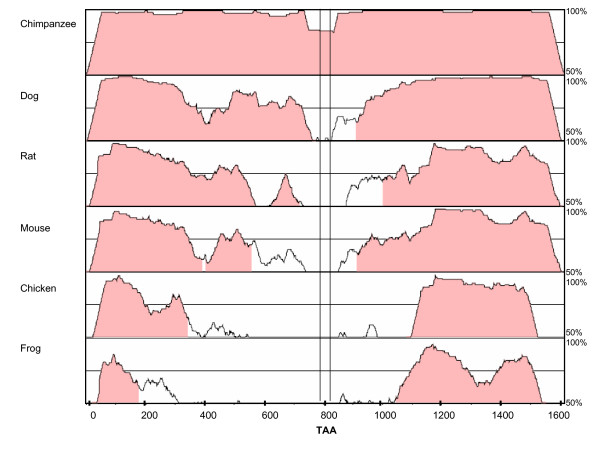
**Evolutionary conservation of the *GRIK2 *3'UTR region**. Plots comparing chimpanzee, dog, rat, mouse, chicken and frog orthologue sequences to the conserved 3'UTR region of human *GRIK2 *were generated using mVISTA [34-36]. For the human sequence, we chose chr6: 102623080–102624690, comprising 1610 bp sequence downstream from the stop codon, based upon the report of a polyadenylation signal at 1547 bp after the stop codon [29]. The relative location of the polymorphic TAA repeat within the human sequence is shown between vertical lines (from 787 to 828bp). Plots show % identity over a 100 bp window (vertical axis) of chimpanzee (chr5 (Nov 2003): 104613747-104615339), dog (chr12 (July 2004): 62913564-62915139), rat (chr20 (June 2003): 53258651-53260220), mouse (chr10 (May 2004): 49099305-49100874), chicken (chr3 (Feb 2004): 68531473-68532948), and frog (*Xenopus tropicalis*) (Scaffold-599 (Oct 2004): 27668-29079), respectively, relative to the human sequence (horizontal axis) [18].

The 2 Mb genomic region downstream from *GRIK2 *shows no known genes in the March 2006 (hg18) assembly of the human genome sequence [18]. We tested the nearest polymorphic microsatellites, ~1 Mb from the *GRIK2 *3'UTR. The presence of a 149 allele for *D6S1580 *(AFM183YH4) in GUS3225, GUS17507, and GUS17508 left open the possibility of a common origin for this ~1 Mb segment. To narrow this possibility to *GRIK2 *itself, we next sequenced the *GRIK2 *3'UTR region and the 3' portion of intron 16 in the same eight individuals, seeking additional polymorphisms that would help to delineate a potential common ancestral origin for the 3' end of the gene. Upstream from exon 17, we detected three known SNPs: rs2852620, rs1034254, and rs12198351. Though Paschen et al. provided evidence that the TAA repeat is present in the 3'UTR, the full extent of the latter has not been well characterized and is not completely represented in current genome database annotations [19]. Consequently, we examined a segment of ~2 kb downstream from the TAA termination codon of *GRIK2*. We chose this segment based on sequence similarity with *GRIK2 *orthologues from Xenopus, chicken, mouse, rat, dog and chimp (Figure 2) and its presence in mouse and rat *Grik2 *mRNAs. We found two novel SNPs, T>C at 248 bp and T>C at 617 bp 3' to the TAA repeat, which have been designated rs28383483 and rs28383484, respectively (Figure 1).

To simplify haplotype determination across the *GRIK2 *gene, we segregated the individual chromosome 6s from GUS3225 and GUS17508 in hybrid cell lines. As no lymphoblast cell line was available for GUS17507, we also relied on genotyping two siblings of this individual in an attempt to limit possible haplotypes. We typed these hybrid lines and GUS17507 relatives for the TAA repeat and the two new 3'UTR polymorphisms, and we retyped selected polymorphisms reported above to directly confirm local haplotypes.

**Figure 3:**
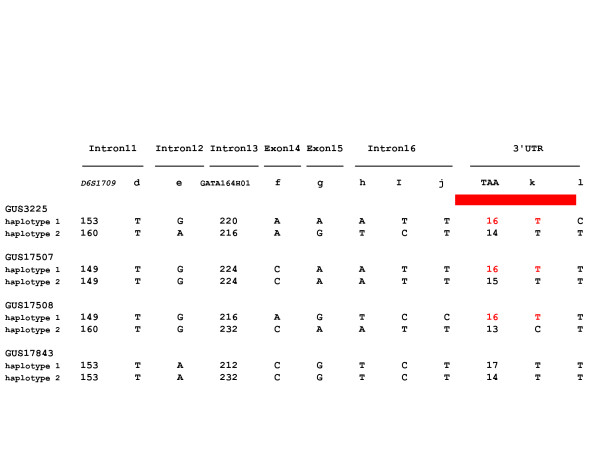
***GRIK2 *3' haplotypes in HD patients with earlier than expected onset**. Haplotypes determined for microsatellite and SNP (labeled as in Figure 1) markers from *GRIK2 *exon 11 through the 3'UTR are shown for the four HD patients with earlier than expected onset, three possessing a 16 TAA allele at *D6S1028 *and one possessing a rare 17 TAA allele. The 16 TAA allele chromosomes are distinguishable by other polymorphisms except in the fully sequenced 2.3 kb region between rs12198351 and rs28383484 (denoted by a red bar above this region of the haplotype) suggesting that the TAA allele is responsible for the modifier effect.

The haplotypes associated with the three 16 TAA alleles and one 17 TAA repeat allele from the individuals with earlier than expected onset of HD are shown in Figure 3. Phasing of the alleles for the previously analyzed polymorphisms revealed that, on the chromosome associated with younger HD onset, all individuals differ for the haplotype of SNPs at exons 14 and 15. This suggests that any potentially shared region responsible for the modifier effect would lie 3' to exon 15. Further, GUS3225 and GUS17507 differ from GUS17508 for the SNPs in intron 16, suggesting that any ancestrally shared region would lie 3' to rs12198351, 1.7 kb upstream from the TAA repeat. However, GUS3225 differs on the 16 TAA chromosome from the other individuals at SNP rs28383484, suggesting that any ancestrally shared region would lie 5' to this site, 617 bp downstream of the TAA repeat. Within the segment between SNPs rs12198351 and rs28383484, which we sequenced in its entirety, only the TAA repeat allele consistently distinguishes these chromosomes from chromosomes not associated with younger than expected onset of HD, suggesting that the length of the TAA allele itself is responsible for the modifier effect.

## Discussion

*GRIK2*, encoding the GluR6 subunit of the kainate receptor, is abundantly expressed in the caudate nucleus, cortex, hippocampus and cerebellum in human. Kainate receptors are one of three subtypes of ionotropic receptors for the excitatory amino acid transmitter L-glutamate. They both mediate excitatory synaptic transmission at primary afferent synapses and modulate presynaptic neurotransmitter release [20]. Interaction of excitatory amino acids with their receptors can potentially cause toxicity that upsets Ca^++ ^homeostasis and damages energy metabolism, and such 'excitotoxicity' due to glutamate has long been postulated to be involved in HD pathogenesis [21,22]. However, a recent analysis of systemic 3-nitropropionic acid induced striatal lesions in *Grik2*^-/- ^mice showed no difference in final histopathology or motor impairment compared with similarly treated wild-type mice, suggesting that striatal GluR6 kainate receptors do not play a critical role in determining neuronal pathology [23]. In this experiment, *Grik2*^-/- ^mice did display motor symptoms earlier than wild-type mice, despite the lack of difference in neuropathology, suggesting that the GluR6 kainate receptors may have played some modulatory role in synaptic transmission rather than mediating excitotoxicity.

There is abundant evidence that genetic modifiers of HD pathogenesis exist, from studies performed before the *HD *gene was cloned through studies performed more recently with the benefit of molecular definition of the HD mutation [5-16,24,25]. *GRIK2 *is currently the only genetic modifier of HD that has been specifically identified and confirmed in more than two independent studies [6,7,11-13]. Our findings indicate that 'modifier' chromosomes did not have a functional coding sequence difference that directly alters GluR6. Rather, they shared the 16 TAA allele but had different haplotypes for SNPs within a 2.3 kb region encompassing the polymorphic repeat, suggesting that the TAA allele itself, rather than a linked polymorphism, is responsible for the modifier effect. Though the effect may be peculiar to 16 TAA alleles, we cannot exclude that it is a length-dependent effect encompassing both 16 TAA and longer alleles, as there are too few 17 TAA alleles to test for a statistically significant modifier effect. However, the very early onset of GUS17843 suggests that 17 TAA alleles may also have the potential to modify neurologic onset in HD.

It is likely that the 3' UTR TAA repeat modifier effect acts at the level of the *GRIK2 *mRNA, with multiple opportunities for allelic alterations in regulation beyond considerations of translational efficiency and stability. Indeed, with the exception of the immediate vicinity of the TAA repeat, the 3'UTR of *GRIK2 *is remarkably conserved across species, suggesting that it has an important regulatory role (Figure 2). *GRIK2 *displays alternative splicing of exon 16 that leads to two major splice variants encoding proteins with different carboxyl-termini. Two other minor alternative splice forms, one skipping exon 12 (predicting a truncated protein) and one in which a cryptic splice site in exon 11 joins to exon 13 (predicting a protein lacking the first two of four transmembrane domains), have been described. Moreover, three major A to I RNA editing sites that can affect ion permeability of the receptor due to resulting amino acid substitutions I>V, Y>C and Q>R, respectively are located in exons 11 (2) and 12. Fully unedited receptors at the Q/R site exhibit higher relative calcium permeability and higher channel conductance as compared to edited receptors [26-28]. Interestingly, two alternative transcripts differing in the length of the 3'UTR have been reported, only one of which includes the TAA repeat [19,29]. The shorter mRNA uses a polyadenylation signal located 92 bases after the stop codon, upstream of the TAA repeat while the longer mRNA extends beyond a putative polyadenylation signal 1547 bases downstream of the stop codon, 3' to the TAA repeat [29]. Thus, numerous opportunities exist for the TAA repeat polymorphism to potentially modify HD onset by virtue of an allelic regulatory effect on the production, polyadenylation status, alternative splicing, subcellular targeting, editing or stability of the *GRIK2 *mRNA. The aforementioned *Grik2*^-/- ^mouse experiments also indicate that an effect on age at neurologic onset may not necessarily be mediated by excitotoxicity, but could occur due to an independent effect on motor function. The fact that *GRIK2 *is capable of modifying the pathogenic process in HD sufficiently to have a noticeable impact on onset age indicates that it is a potential target for development of an effective therapeutic intervention. However, it will be necessary to delineate of the precise basis for the *GRIK2 *modifier effect in order to effectively undertake a search for chemical compounds that delay, rather than hasten HD onset.

## Conclusion

*GRIK2 *acts as a modifier of HD pathogenesis, not through a coding sequence polymorphism that changes the structure of the GluR6 protein, but rather through a TAA repeat polymorphism in the 3'UTR. Translating this knowledge into a treatment to delay HD pathogenesis will first require definition of the functional consequences of the TAA repeat, possibly involving altered regulation of *GRIK2 *mRNA.

## Methods

### Patient DNA and cell lines

After informed consent was obtained in accord with institutional review board (IRB) policies, blood samples were obtained from patients and family members. Standard methods were used for extraction of DNA and, in some cases, generation of lymphoblastoid cell lines.

### Statistical analysis

Variability in onset age attributable to the CAG repeat number was controlled by linear regression using the logarithmic transformed age at onset as the dependent variable and *GRIK2 *TAA, normal CAG repeat, *HD *CAG repeat, and the product term between normal CAG repeat and *HD *CAG repeat as independent variables [30-32].

### *GRIK2 *sequencing

We amplified 17 coding exons of *GRIK2 *(NM_021956) and 1991 base pairs downstream from the stop codon, with primers located in flanking intron and UTR sequences. Amplification was performed in a standard 30 μl PCR reaction (in 10 mM Tris, pH 8.9, 50 mM KCl, 1.5–2.5 mM MgCl_2_, 10 pmol of each primer and 200 μM of each dNTP, including 50–100 ng DNA and 1U Taq polymerase (Invitrogen) using an MJ Research PT-100 with the following conditions: 94°C, 4 min, 30 X (94°C, 45s; 50–60°C, 45s; 72°C, 1 min), 72°C, 10 min. PCR products were analyzed by direct sequencing by using BigDye Terminator Cycle Sequencing Kit (Applied Biosystems) on an ABI 377/XL DNA Sequencer (Perkin-Elmer).

### Microsatellite genotyping

PCR used 30 ng/ul genomic DNA; 0.1–1.0 ul (4 uM Stock Markers) of each dye-labeled primer pair (Research Genetics and custom oligos from Applied Biosystems); 0.1 ul (5 units/ul) Ampli *Taq *Gold DNA polymerase (Applied Biosystems); 0.75 ul10x GeneAmp PCR buffer II (Applied Biosystems); 0.75 ul dNTP mix (2.5 mM, Amersham); 0.75 MgCl_2 _(25 mM); and dH_2_0 to 7.5 ul, and was executed in the MJ Research DNA Engine tetrad with the following conditions: 95°C, 12 min;10X (94°C, 30s, 58°C, 30s; 72°C, 60s); 25X (89°C, 30s; 55°C, 30s; 72°C, 60s); 72°C, 40 min. Except for *D6S1555, D6S1028, D6S449, D6S1580 *which cycled: 95°C, 5 min; 35X (94°C, 30s; 50°C, 30s; 72°C, 45s) 72°C, 10 min. *D6S301 *cycled: 95°C, 5 min; 53X (94°C, 30s; 54°C, 30s; 72°C, 45s) 72°C, 10 min. PCR products were run on a 5% Gene_PAGE PLUS (Amresco) gel on an ABI 377/XL DNA Sequencer for three hours, with a size standard GeneScan-500 TAMRA) in each lane to ensure lane-to-lane and gel-to-gel size-calling consistency. Data were analyzed with ABI's GeneScan Version 3.1 and Genotyper version 2.5 software.

## Abbreviations

HD, Huntington's disease; UTR, untranslated region.

## Authors' contributions

WZ carried out the molecular genetic studies, participated in the sequence analysis and drafted the manuscript. TG and MH carried out the sequencing and microsatellite marker analysis. LD performed the statistical analysis. RHM participated in the design of the study. MEM and JFG conceived of the study, and participated in its design and coordination and helped to draft the manuscript. All authors read and approved the final manuscript.

## Figures and Tables

**Table 1:** Selected HD patients with similar CAG repeat size but different onset ages

**Patient**	***HD *CAG Repeat**	**Age of onset (yrs)**	***GRIK2 *TAA repeat**
			
	**HD allele/normal allele**		**allele 1/allele2**
**Early onset**			

GUS3225	46/18	28	14/16
GUS17507	45/26	33	15/16
GUS17508	47/16	29	13/16
GUS17843	46/23	33	14/17

**Typical onset**			

GUS2433	46/19	57	14/14
GUS18027	47/21	54	14/15
GUS20708	46/17	50	12/15
GUS21069	45/17	58	15/15

**Table 2:** Genotypes in the *GRIK2 *region in selected HD patients

		**Earlier**	**Age of**	**Onset**		**Normal**	**Age of**	**Onset**
		
	**GUS3225**	**GUS17507**	**GUS17508**	**GUS17843**	**GUS2433**	**GUS18027**	**GUS20708**	**GUS21069**
**SNPs**								
rs2786251 (Intron 5)	G/G	G/G	G/G	G/G	G/G	G/G	A/A	G/G
rs6922753 (Intron 5)	C/C	C/C	C/C	C/C	C/C	C/C	G/G	C/C
rs6922753 (Intron 7)	T/T	T/C	T/T	T/T	T/C	T/T	T/C	T/T
rs2518283 (Intron 11)	T/T	T/T	T/T	T/T	T/T	T/T	T/C	T/T
rs2243355 (Intron 12)	G/A	G/G	G/G	A/A	G/A	G/A	G/G	G/G
rs3213607 (Exon 14)	A/A	C/C	C/A	C/C	C/C	C/A	C/C	C/A
rs2227283 (Exon 15)	G/A	A/A	G/A	G/G	G/A	G/G	G/G	G/A
rs2852620 (Intron 16)	A/T	A/A	A/T	T/T	A/T	T/T	T/T	A/T
rs1034254 (Intron 16)	T/C	T/T	T/C	C/C	T/C	C/C	C/C	T/C
rs12198351 (Intron 16)	C/C	G/G	C/C	C/C	G/C	C/C	C/C	C/C
rs28383483 (3'UTR)	T/T	T/T	T/C	T/T	T/T	T/T	T/T	T/T
rs28383484 (3'UTR)	T/C	T/T	T/T	T/T	T/T	T/T	T/T	C/C
**Microsatellites**								
*D6S468*	152/154	154/156	152/156	150/158	156/160	156/158	154/156	152/158
*D6S1555*	290/294	296/296	294/294	290/292	294/296	290/296	294/296	290/296
*D6S449*	197/197	197/197	197/197	195/197	197/197	197/197	191/191	No data
*D6S1709*	153/160	149/149	149/160	153/153	149/153	153/153	153/168	149/160
GATA164H01	216/220	224/224	216/232	212/232	224/224	220/224	220/232	220/224
*D6S1028 *(TAA)	14/16	15/16	13/16	14/17	14/14	14/15	12/15	15/15
*D6S1580*	149/149	149/168	149/160	149/157	160/162	149/168	149/153	149/162
*D6S1679*	253/253	249/253	243/244	244/253	242/244	242/242	243/247	244/244
*D6S301*	228/228	228/228	246/248	228/248	242/248	244/244	236/246	248/248
